# 3D imaging and single-cell analysis reveal cellular heterogeneity of lymphatic valve endothelial cell types

**DOI:** 10.1016/j.isci.2025.113841

**Published:** 2025-10-24

**Authors:** Emmanuelle Marchaud, Renaud Morin, Jason S. Iacovoni, Tangra Draia-Nicolau, Aurélie Gomes, Marine Norlund, Pascale Bernes-Lasserre, Jean-Michel Lagarde, Anne-Catherine Prats, Barbara Garmy-Susini, Anne Bouloumie, Anaïs Briot, Florence Tatin

**Affiliations:** 1 University of Toulouse, INSERM, I2MC, Toulouse, France; 2 IMACTIV3D, SAS, Centre Pierre Potier, Toulouse, France

**Keywords:** Vascular anatomy, Lymphology, Integrative aspects of cell biology, Transcriptomics

## Abstract

Lymphatic vasculature has specialized vessels to support unidirectional interstitial fluid transport and immune surveillance. Although the lymphatic system is a critical actor in skin homeostasis, the spatial organization of the lymphatic network from the reticular dermis to the hypodermal adipose tissue remains elusive. Here, we used 3D imaging to determine the lymphatic architecture within the adult skin microenvironment. We showed that lymphatic vasculature forms a highly defined hierarchical organization from the dermal capillary to collecting lymphatic vessels located exclusively in the dermal adipose layer. Further examination revealed multiple lymphatic valves located at the intersection between capillaries and precollecting vessels. In agreement with our 3D imaging, single-cell transcriptomics uncovered two clusters of lymphatic valve endothelial cells with distinct gene signatures and signaling pathways. In summary, we provide a better understanding of the spatial organization and heterogeneity of lymphatic endothelial cells that underline the importance of lymphatic valve in adult skin.

## Highlights

• 3D imaging reveals a hierarchical lymphatic network in adult murine skin
• Lymphatic valves are enriched in lymphatic capillaries in reticular dermis
• Single-cell analysis identifies two distinct subpopulations of lymphatic valves
• Valve clusters share gene signatures across skin and mesenteric tissues

## Graphical abstract

**Figure:**
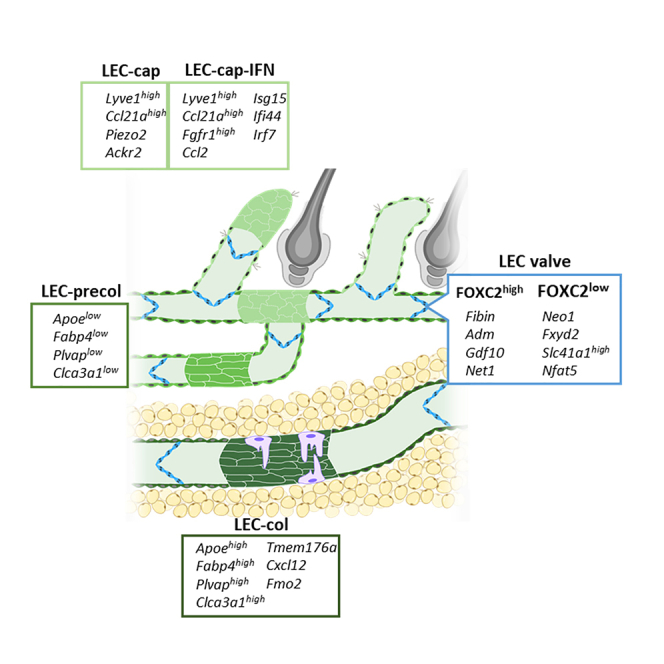

## Introduction

The lymphatic system is a critical actor for skin homeostasis, participating in fluid balance and immune surveillance. Previous studies on ear skin characterized the lymphatic vessel network based on morphology, protein expression, and cell-cell junction organization. Initial lymphatic capillaries are blind-ended lymphatic vessels with specialized button-like cell-cell junctions, enabling entry of fluids, macromolecules, and immune cells, which are then transported by collecting lymphatic vessels.^1^ Lymphatic capillaries express higher protein levels of LYVE1, REELIN, and CCL21 chemokine, while collecting vessels express markers such as FOXP2 and ACKR4.^2^^,^^3^^,^^4^ Common features of precollecting and collecting vessels include the presence of bi-leaflet intraluminal valves and continuous adherens junctions, namely zipper-like junctions, while only lymphatic collecting vessels are covered by smooth muscle cells.^1^^,^^2^^,^^5^ Intraluminal lymphatic valves are characterized by a high level of the transcription factor PROX1 and ensure unidirectional fluid flow in the lymphatic network.^6^^,^^7^ Importantly, defects in lymphatic valve morphogenesis, as well as dysfunctional valves, impair lymphatic drainage and are associated with lymphedema, obesity, and inflammatory bowel disease.^8^^,^^9^^,^^10^ Recently, single-cell studies on ear skin and mesenteries revealed the diversity of lymphatic subtypes with specific gene signatures for lymphatic capillaries and collecting lymphatic vessels.^11^^,^^12^^,^^13^ In addition, specialized lymphatic capillary subpopulations were found to regulate immune responses in ear skin, including the expression of *Ptx3*, *Ackr2*, and *Itih5*.^13^ Similarly, a *Cldn11*, *Prox1*^*high*^, *Naftc1*^*high*^, and *Gja4*^*high*^ gene signature indicates lymphatic valve endothelial cells.^11^^,^^12^^,^^13^ Therefore, in-depth analysis of lymphatic endothelial cell (LEC) subtypes in different organs will allow the functional characterization of specific LEC subpopulations.^14^ Given that lymphatic vasculature has an organ-specific organization and function,^14^ we aimed to investigate the structural organization of the lymphatic vasculature in the murine forelimb and dorsal skin microenvironment, which represent relevant models exhibiting well-defined architectures composed of functionally distinct layers of specialized cells, ranging from the epidermis to the dermal adipose layer.^15^ The role of skin lymphatic vasculature is of particular importance in human health and disease. Dysfunction of lymphatic vasculature is linked to lymphedema and is now recognized to worsen the pathological effects of obesity and type II diabetes.^16^^,^^17^ Using 3D imaging with light-sheet microscopy, we revealed that lymphatic vasculature has a highly hierarchical organization with distinct subpopulations of lymphatic vessels in close association with the skin microenvironment. Interestingly, our work showed the presence of numerous lymphatic valves strategically located within the lymphatic network, with the highest number at the intersection between capillaries and precollecting vessels. Our single-cell analysis indicates 6 subsets of LECs, including two clusters of lymphatic valve endothelial cells with distinct gene signatures and signaling pathways. In addition, the comparison of our dataset with published single-cell transcriptomics from mesenteric lymphatic vessels indicates that these two lymphatic valve clusters are a common feature of lymphatic valves. Our dataset provides a comprehensive structural and molecular atlas of lymphatic subpopulations and valuable information to further decipher lymphatic diversity and function in skin microenvironments.

## Results

### Lymphatic vessels form a highly organized hierarchical architecture in adult skin

**Figure 1:**
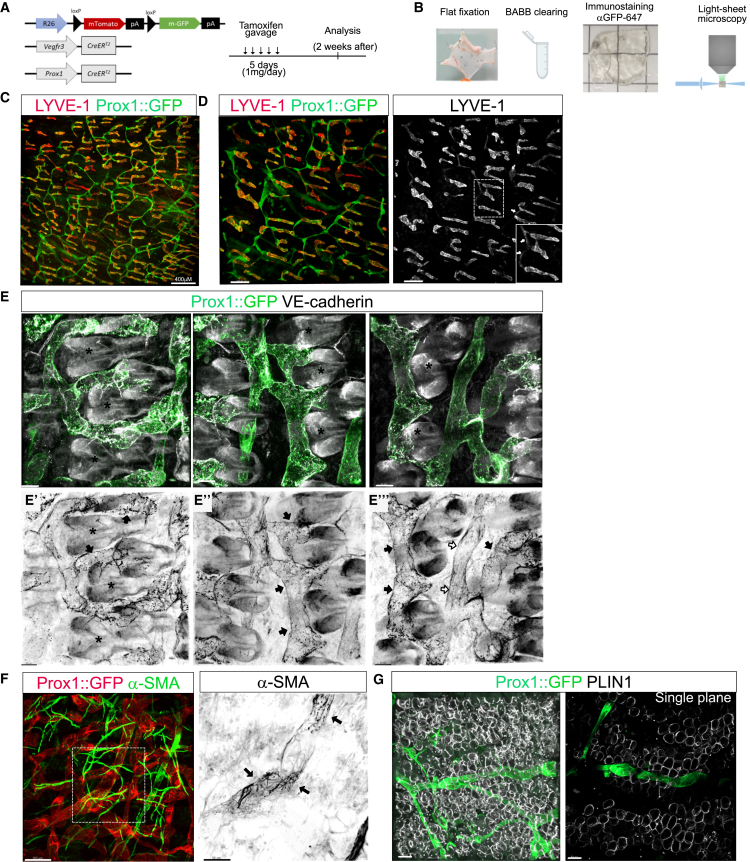
Adult skin shows a hierarchical lymphatic network (A) Schematic representation of the breeding of *Vegfr3-creER*^*T2*^ or *Prox1-creER*^*T2*^ with *R26-mTmG* mice and the administration of tamoxifen, leading to the expression of GFP by target cells. (B) Protocol for whole-mount light-sheet imaging on skin. Skin is collected and flat-fixed for 24 h. The skin is immunostained with an anti-GFP antibody coupled with Alexa Fluor 647 nm and cleared using the BABB clearing protocol. (C) LYVE1 immunostaining on dorsal skin allows visualization of lymphatic capillaries. Scale bar: 400 μm. (D) Magnified view of the lymphatic network in dorsal skin with the connection of LYVE1-positive capillaries. Arrows show lymphatic vessels with lower LYVE1 expression. Scale bar: 200 μm. (E) VE-cadherin staining in lymphatic capillaries, LYVE1^low^ precollecting vessels around hair follicles (E′ and E″, black arrows) and LYVE1^neg^ precollecting vessels (E‴, white arrows). Scale bar: 30 μm. (F) α-SMA (alpha-smooth muscle actin) immunostaining (in green) and GFP-positive lymphatic vessels (shown in red) reveal the presence of smooth muscle cells scattered on collecting lymphatic vessels Scale bar: 100 μm. A magnified image of α-SMA-positive cells is shown. Scale bar: 50 μm. (G) Collecting lymphatic vessels are associated with mature adipocytes labeled by perilipin staining. Lymphatic vessels are in the same plane as the adipocytes. Scale bar: 50 μm.

The reticular dermis is a layer enriched in extracellular matrix components and the dermal adipose tissue closely attached to the dermis contributes to skin homeostasis and is an active participant in the battle against infection or injury.^18^^,^^19^ In dorsal skin, the layer of dermal adipose tissue is physically separated from subcutaneous adipose tissue by a fibrous layer of muscle, the *panniculus carnosus,* absent in forelimb skin (see Figure S1). In order to visualize the lymphatic vasculature in murine skin, we first performed immunodetection on paraffin cross-sections of LYVE1 and PODOPLANIN (PDPN) to specifically identify lymphatic vessels and PERILIPIN1 (PLIN1) to label adipocytes on paraffin cross-sections (see Figure S1). This approach permits detection of lymphatic vessels in the dermis of forelimb skin and dorsal skin but precludes visualization and characterization of the entire lymphatic network extending further into the tissue. Therefore, we took advantage of light-sheet microscopy, allowing deep 3D imaging of thick samples, and reporter mouse models with GFP expression targeting the lymphatic vasculature under control of either the Vegfr3 promoter (*VEGFR3-CreER*^*T2*^*; mTmG)* or the Prox1 promotor (*Prox1CreER*^*T2*^*; mTmG)* (Figure 1A). For light-sheet imaging, skin samples were cleared with BABB solution (benzyl alcohol and benzyl benzoate), which is known to quench GFP and Tomato fluorescence. To circumvent this, we performed whole-mount skin immunostaining with anti-GFP conjugated to Alexa Fluor 647 dye to avoid sample autofluorescence by the 488 nm excitation wavelength (Figure 1B). Analysis of forelimb skin and its associated brachial adipose tissue revealed that the majority of the lymphatic network is located in skin with few lymphatic collecting vessels present in the deeper subcutaneous adipose tissue. We were thus able to follow a collecting vessel with lymphatic valves taking its origin in the dermis and reaching the brachial lymph node (see Figure S2A, framed by cyan dotted lines). Most lymphatic vessels are spatially restricted to the dermis (see Figure S2B; Videos S1 and S2). Analysis of the dorsal skin confirms a similar organization of the lymphatic network, although the presence of a highly fluorescent muscle layer limits the visualization of the lymphatic vasculature (see Figure S2C). Therefore, we selected specific z stacks to visualize the lymphatic network in dorsal skin (see Figure S2C and Video S3). We showed that lymphatic capillaries, which selectively expressed a high level of LYVE1 (LYVE1^high^), are connected to lymphatic vessels, which have at most a partial expression of LYVE1 (LYVE1^low^), suggesting that this network represents precollecting vessels (Figures 1C and 1D). Thereafter, we investigated the junctional organization of lymphatic vessels using different Z positions of VE-cadherin immunostaining by confocal microscopy. Lymphatic capillaries and precollecting vessels in close proximity to hair follicles appear to have discontinuous button junctions (black arrows, Figures 1E’–1E”) in contrast to continuous junctions observed in precollecting vessels located deeper in the dermis (white arrows, Figure 1E’’’). We next examined the localization of lymphatic vessels in the dermal adipose layer. Unlike capillaries and precollecting vessels in dermis, collecting lymphatic vessels are partially covered by smooth muscle cells and are embedded between dermal adipocytes expressing PLIN1 (Figures 1F and 1G). This dermal adipose layer in developing skin is surrounded by the reticulum interstitium, which is an anatomical distinct layer enriched in extracellular matrix and multipotent stromal progenitors cells positive for DDP4 (Dipeptidyl peptidase-4).^20^ Lymphatic collecting vessels (GFP+) reside in the dermal adipose layer right above the reticulum interstitium in forelimb skin, while they are separated by the muscle layer in dorsal skin (see Figures S2D–S2F). Overall, we defined a stratification of capillaries, precollecting and collecting lymphatic vessels that correlate with the dermal microenvironment.

Video S1. The 3D surface reconstruction of the entire lymphatic vasculature in forelimb skin, related to Figure S2 3D segmentation of the lymphatic network in the skin of *Prox1-creER*^*T2*^*; mTmG* mouse acquired with a light-sheet microscope (2,5× objective) after GFP staining and BABB clearing. The acquisition is 1,700 × 1,700 × 370 μm. The animation turns around the longitudinal axis of the dermis. The lymphatic network is really dense in the skin with blind-ended lymphatic capillaries oriented toward the epidermis. The software ImageJ was used for the 3D animation.

Video S2. Visualization of the deeper lymphatic collecting vessels in forelimb skin, related to Figure S2 3D segmentation of the entire lymphatic network in the skin of *Prox1-creER*^*T2*^*; mTmG* mice acquired with a light-sheet microscope (2,5× objective) after GFP staining and BABB clearing. The acquisition is 1,700 × 1,700 × 370 μm. The animation turns around the transversal plane with the epidermis at the bottom and the dermal white adipose tissue at the top. The blind-ended lymphatic capillaries are oriented toward the epidermis and the lymphatic collecting vessels form different layers at different depths within the skin that are parallel to the epidermis. The software ImageJ was used for the 3D animation.

Video S3. z Stack imaging of the lymphatic vasculature in mouse dorsal skin, related to Figure S2 z stack of the lymphatic network in dorsal skin of *Prox1-creER*^*T2*^*; mTmG* mice acquired with a light-sheet microscope (2,5× objective). The acquisition is 3.51 × 3.51 × 2.07 mm. The software ImageJ was used for the 3D animation starting from the muscle layer toward the epidermis.

### Lymphatic valves are a predominant feature of the dermal precollecting network

**Figure 2:**
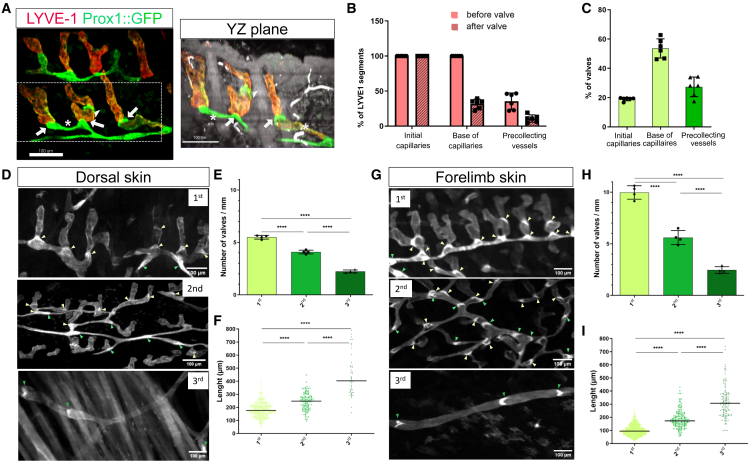
Localization of lymphatic valves across the lymphatic network (A) Magnified view of lymphatic capillaries with LYVE1 immunostaining. Arrows show lymphatic valves at the junctions between capillaries and precollecting vessels with partial LYVE1 expression (LYVE1^low^). Arrowheads show lymphatic valves in a single capillary. YZ plane view of (A) retaining the autofluorescence of the epidermis and hair follicles. LYVE1^low^ precollecting vessels (white stars) mark the junction between two capillaries separated by a hair follicle. Scale bar: 100 μm. (B) Quantification of LYVE1 expression depending upon valve position. (C) Percent of valves depending on their location within the lymphatic network from 3 distinct experiments. Data (B and C) are from 3 distinct experiments (*n* = 3 mice, 6 crops of 1 mm^3^). (D–F) Position of lymphatic valves in dorsal skin within the first network (light green), second network (green), and third network (dark green) Scale bar: 100 μm. (E) Quantification of the number of valves reported to the length of the network layer in dorsal skin. (F) Distances between two valves in the dorsal lymphatic network. (G–I) Position of lymphatic valves in forelimb skin within the first network (light green), second network (green), and third network (dark green) Scale bar: 100 μm. (H) Quantification of the number of valves reported to the length of the network layer in forelimb skin. (I) Distances between two valves in the forelimb lymphatic network. Data (E, F, H, and I) are from 4 distinct experiments, plots (C, E, and H) show means ± SD, and plots (F) and (I) show medians. ∗∗∗∗*p* < 0.0001, one-way ANOVA with Bonferroni correction.

We noticed that lymphatic capillaries are similarly oriented with respect to the skin surface as measured by three angles of rotation (roll, pitch, and yaw) (Figure 1C, also see Figures S3A and S3B). These results corroborate with previously published work demonstrating a close association of lymphatic capillaries with hair follicles.^21^^,^^22^ We performed an image segmentation based on the lymphatic architecture assuming that “open segments” represent initial lymphatic capillaries (red line connected to cyan nodes), while “connected segments” represent other lymphatic vessel types (blue line connected to yellow nodes) (see Figure S3C). Initial lymphatic capillaries, representing around 30% of the total density of the lymphatic network, show variable length (see Figures S3D and S3E). Importantly, a large proportion of intraluminal lymphatic valves are observed, a feature we were not able to evaluate on cross-sections (see Figures S3F–S3H). Intraluminal lymphatic valves are easily identified by the presence of dense GFP expression in *Prox1creER*^*T2*^*; mTmG* mice, shown in the segmented image as green dots in Figure S3G and segmented in red in Figure S3H. Therefore, we decided to investigate the position and occurrence of lymphatic valves. We visualized some lymphatic valves within capillaries, defined by a strong LYVE1 expression (LYVE1^high^) and a similar orientation in the tissue (arrowheads, Figure 2A). In addition, we consistently found a lymphatic valve at the base of lymphatic capillaries linking to LYVE1^low^ precollecting lymphatic vessels (arrows, Figure 2A). Our quantification showed that downregulation of LYVE1 always occurs downstream of the valve located at the base of capillaries (arrows, Figures 2A and 2B). Overall, these results suggest that the presence of a lymphatic valve alone does not influence LYVE1 expression. However, their position in the lymphatic network (in our case, at the base of the oriented lymphatic capillaries) defines the occurrence of precollecting vessels, which are most often surrounding hair follicles (Star, Figure 2A). Surprisingly, the proportion of lymphatic valves located within and at the base of capillaries represents more than 60% of lymphatic valves in the lymphatic network (Figure 2C). To better quantify the occurrence of lymphatic valves in forelimb and dorsal skin, we discriminated 3 types of lymphatic networks depending on their Z position in the skin. The first network defined lymphatic capillaries and LYVE1^low^ precollecting vessels directly connected to capillaries (around 140 μm depth) (Figures 2D and 2G). The second network corresponds to highly branched precollecting vessels running parallel to the skin surface (around 160-180 μm depth) connected to a third network located deeper in dermal adipose layer, the collecting lymphatic vessels (around 240 μm depth). Noticeably, we found that precollecting vessels that are connected to capillaries have a high number of lymphatic valves with a short distance between them suggesting that valves may actively support lymph drainage from capillaries to precollecting vessels (Figures 2E and 2H). As the number of lymphatic valves decreases through the lymphatic network, the distance between lymphatic valves increases (Figures 2F and 2I). The forelimb and dorsal skin share a similar lymphatic architecture; however, we unexpectedly found that the precollecting network in forelimb skin contains nearly twice as many valves, with a shorter distance between them (Figures 2E–2H and 2F–2I). We found a higher number of lymphatic valves in the forelimb skin (∼210 valves/mm^3^) compared to the dorsal skin (∼100 valves/mm^3^), in agreement with a tissue-specific organization of lymphatic vessels.

### Single-cell analysis reveals two lymphatic valve subpopulations

**Figure 3:**
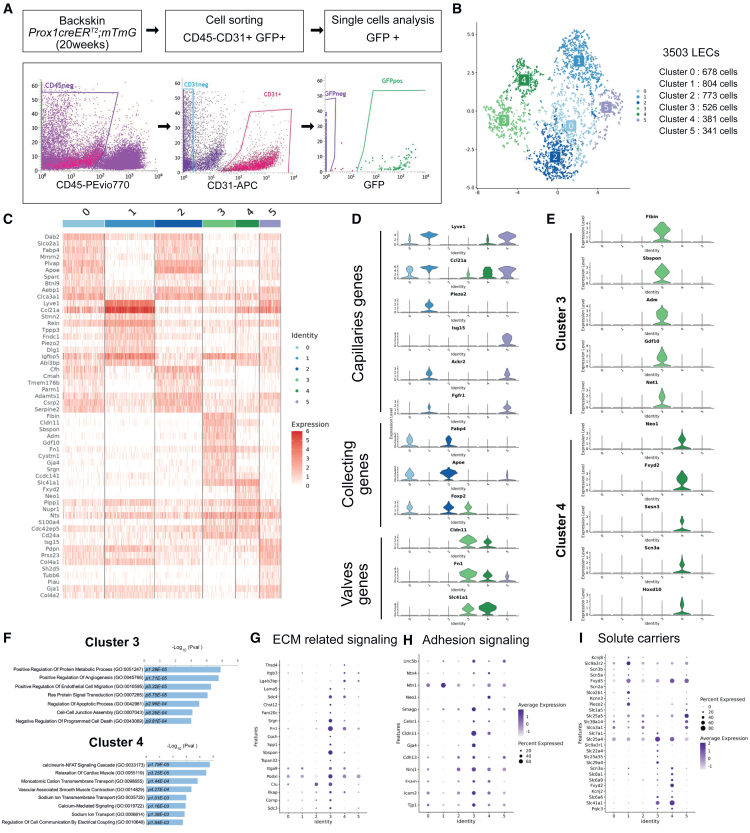
Single-cell RNA sequencing reveals specific subsets of lymphatic valve endothelial cells (A) Schematic showing the experimental design and FACS analysis to isolate GFP-positive cells for single-cell analysis. (B) UMAP plot of the 6 identified LEC clusters. (C) Heatmap of top 10 per cluster differentially expressed genes. (D) Violin plots of known markers to differentiate clusters belonging to lymphatic capillaries, collecting vessels and lymphatic valves. (E) Violin plot of genes differentially expressed in the two clusters of lymphatic valves. (F) Bar plots of GO categories that were enriched using genes upregulated in lymphatic valve clusters 3 and 4. (G–I) Dot plots of selected genes that discriminate valve clusters related to genes from pathways related to extracellular matrix signaling (G), adhesion signaling (H), and solute carriers (I). The color code indicates scaled average expression level. Dot size indicates the percent of cells expressing the gene of interest.

To assess LEC heterogeneity, we performed single-cell transcriptomics analysis (scRNA-seq) on native LECs isolated by cell sorting defined as CD45^−^, CD31^+^, and GFP+ from the reporter *Prox1creER*^*T2*^*; mTmG* mouse model (Figure 3A). A total of 3,503 high-quality LECs expressing *Flt4* and *Prox1* were clustered into 6 different subpopulations (Figure 3B). Heatmap showing normalized expression of top 10 ranked markers per cluster shows distinct gene signatures (Figure 3C). Based on gene expression of known LEC markers, we annotated lymphatic capillaries (cluster 1 and 5 expressing *Lyve1*, *Ccl21a*, and *Reln*), collecting vessels (cluster 0 and 2 expressing *Fabp4*, *ApoE*, and *Foxp2*) and lymphatic valves (cluster 3 and 4 expressing *Cldn11*, *Fn1*, and *Slc41a*) (Figures 3D and 3E). In our data, we discriminated the presence of an LEC^cap^ cluster, expressing *Lyve1* and *Ccl21a* as well as pro-inflammatory chemokines *Ccl2* and increased expression of interferon-responsive genes (see Figure S4A). Gene Ontology (GO) analysis of cluster 5 marker genes showed enrichment in biological pathways involved in defense response (GO:0051607, GO:0140374) and interferon regulation (GO:0034341, GO:0032481) (Figure S4B). Volcano plot displays the fold change and statistical significance of genes with increased expression in cluster 5 (see Figure S4C). We next examined the molecular profile of collecting vessels in cluster 0 and 2 (see Figure S4D). These two clusters share in common known markers of collecting lymphatic vessels such as *Foxp2*, *Apoe*, *Fabp4*, and *Plvap*.^12^^,^^13^ Additional specific genes involved in metabolic processes such as *Fmo2 and Lsr* or immune signaling such as *Tmem176 and Cxcl12* reveal the identity of collecting vessels. Of particular interest, gene ontology enrichment analysis and functional and predictive protein association networks highlight that collecting LECs are enriched in genes involved in negative regulation of coagulation, especially the thrombin-thrombomodulin complex (*Thbd*, *Procr*, *Vwf*, and *Plvap)* and genes related to complement activation (*Cfh*, *ApoE*, and *Clu*) (see Figures S4E and S4F). Taken together, our data reveal distinct subpopulations of lymphatic capillaries and collecting lymphatic vessels suggesting their participation in innate immune responses that need to be further explored. Additionally, our data revealed two distinct populations of lymphatic valve endothelial cells (Figure 3E; cluster 3 and 4). We found that cluster 3 was defined by the expression of secreted proteins such as *Adm*, *Gdf10*, *Wnt4*, *Hbegf*, and *Nts* (Figure 3E) and by a higher expression of transcription factors *Prox1*, *Foxc1*, and *Foxc2* (Figure S5). Pathway analysis showed an enrichment of genes involved in endothelial cell migration and cell-cell junction assembly for cluster 3 and transport of small molecules for cluster 4 (Figure 3F). More precisely, the subset of valve endothelial cells corresponding to cluster 3 expressed specific extracellular matrix regulators, such as Fibronectin (*Fn1*) Serglycin (*Srgn*), Thrombospondin-5 (Comp), Somatomedin-B, thrombospondin type-1 domain-containing protein (*Sbspon*), and Osteopontin (*Spp1*), suggesting their role in the maintenance of valve leaflet structure. These results are in agreement with previous work that demonstrated the presence of two types of valve endothelial cells with a higher expression of FOXC2 and matrix proteins in valve sinuses compared to cells located in the luminal part of valve leaflet.^23^^,^^24^ Similarly, we showed an enrichment in Netrin/Neogenin/Unc5b signaling as well as both novel adhesion molecules (*Smagp*, *Ninj1*, and *Cdh13*) and previously established ones *Claudin 11* and *ZO1 (Tjp1)* and *Celsr1* (Figure 3H). We also noticed enrichment in solute carriers for specific LEC clusters. Notably, we found that *Slc41a1*, which encodes the sodium-magnesium transporter, is highly expressed in cluster 4 and to a lesser extent in cluster 3. In addition, we found genes related to ion transmembrane transport involved in cardiac conduction and/or contractility such as sodium ion transport (*Scn3a*, *Slc6a6*, and *Slc6a9*), sodium-calcium exchange (*Slc8a1)*, the γ-subunit of sodium-potassium ATPase (*Fxyd2* and *Fxyd5)* and potassium channels (*Kcnj2*). Furthermore, we found that this subset of valve endothelial cells has a higher expression of the transcription factor *NFAT5* (TonEBP), which is activated by osmotic pressure, and *Irx3*, known to modulate gap junction proteins and be associated with heart conduction^25^ (see Figure S5). Taken together, these findings suggest that the transport of osmolytes across the membrane may modulate the potential membrane of this subset of valve endothelial cells, contributing to dynamic contraction-relaxation phases of lymphatic valves.^26^^,^^27^

### Valve subpopulations define a common feature of lymphatic valves

**Figure 4:**
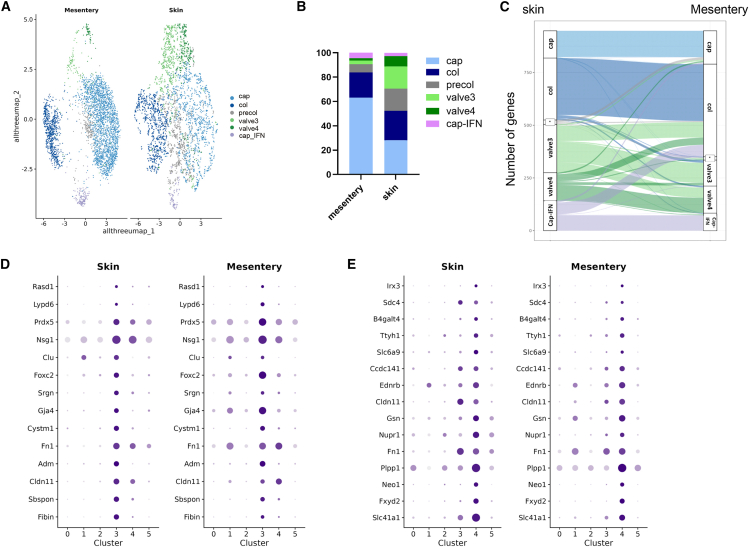
Integrative analysis of lymphatic valve subsets (A) UMAP plot of integrated LEC clusters in mesentery and skin. (B) Proportion of cells assigned to different clusters from mesentery and skin. (C) Alluvial diagram showing the relationship between dermal and mesentery lymphatic cluster markers. ∗ represents precollecting vessels labeled in gray in Figure 4A. Note that this cluster has distinct gene signatures in each tissue. (D and E) Dot plots showing the top marker genes of valve endothelial cell subpopulations in both tissues with the following thresholds: pval_adj < 1e−6, avg_log2FC > 1.0, and (pct.1 – pct.2) > 0.2.

We then wanted to assess whether identified valve clusters and their molecular signatures are tissue specific or a conserved feature of lymphatic valves across different vascular beds. Therefore, we performed a retrospective study of a dataset of LECs isolated from adult lymphatic mesenteries that we compared with our dataset.^12^ UMAP shows the integration of both datasets with a total number of 6 clusters (Figure 4A). In terms of number of cells per cluster, the integrative UMAP (uniform manifold approximation and projection) highlights a higher proportion of cells representing lymphatic valve clusters in our skin dataset whereas fewer cells comprising the 2 distinct valves clusters are found in the mesenteric dataset (Figures 4A and 4B). In agreement with our 3D image quantification, we also noticed a higher number of cells representing precollecting and collecting lymphatic vessels, as well as lymphatic valve endothelial cells in the skin dataset, although the proportion of lymphatic capillaries is higher in the mesenteric dataset (Figure 4B). In addition, we found that the gene signature profile between LEC^cap^, LEC^col^, and LEC^valves^ have a high level of similarity between both tissues as shown by gene expression pattern with greater conservation in the capillaries (LEC^cap^) and collectors (LEC^col^) (Figure 4C). These results suggest that the molecular equipment at the transcriptional level is highly conserved between LEC subsets from both territories. In contrast, LEC^cap−IFN^ and LEC^valves^ clusters present an intermediate profile and share transcript signature with other cell subtypes. For example, few genes related to cellular response to immune signaling expressed in LEC^cap−IFN^ in skin show a lower expression in LEC^col^ cluster in mesentery. Regarding the identity of valve subsets, several genes are shared in common between both LEC^valves^ clusters in skin and in mesentery such as (*Cldn11*, *Slc41a1*, and *Sdc4*) while some of them were also shared in LEC^col^ (such as *Gja4*, *Vangl2*, *Gata2*, and *Foxc1*) (Figures 4C–4E). However, the comparison of the most significant gene markers highlighted a unique gene signature for each lymphatic valve cluster shared by both tissues indicating that both clusters define a distinct and unique subpopulation of lymphatic valve endothelial cells (Figures 4D and 4E).

## Discussion

Despite the importance of lymphatic vasculature in skin homeostasis, the spatial organization of the lymphatic vasculature in adult skin has remained elusive. In this study, we revisited the architecture of lymphatic vasculature based on the anatomy of the adult murine skin to map the lymphatic network. Initial capillaries form a regular and oriented pattern in murine skin in agreement with previous findings showing their association to the hair follicle stem cell niche.^21^^,^^22^ Importantly, 3D imaging revealed the importance of lymphatic valves in forelimb and dorsal skin, which could not be appreciated by conventional skin cross-sections. We determined the presence of multiple lymphatic valves at very short distances apart and located in a regular manner at the intersection between capillaries and precollecting vessels, suggesting that lymphatic valves are of particular importance to propel the lymph and avoid backflow in capillaries. Indeed, lymph flow in lymphatic capillaries is relatively low and increase progressively in contracting lymphatic collectors.^28^^,^^29^ Therefore, lymphatic valves in capillaries may play a crucial role in ensuring the drainage of lymph in the dermis. Surprisingly, only sparse αSMA-positive cells were detected on collecting vessels in dermal adipose tissue indicating that the coverage of smooth muscle cells on collecting vessels vary depending on the tissue.^2^^,^^30^^,^^31^ Our single-cell transcriptomics analysis correlates with our 3D observation as we discriminated two clusters of lymphatic valves, which were further identified in mesenteric lymphatic vessels. Interestingly, we noticed that a peculiar subset of lymphatic valve endothelial cells express genes related to ion transport mediating intracellular changes in calcium, sodium and potassium. This suggests that, in addition to the mechanosensitive transporter Piezo1, critical to valve morphogenesis,^32^^,^^33^ lymphatic valve endothelial cells are properly equipped to regulate osmotic force to dynamically respond to flow. Furthermore, we identified genes related to netrin signaling as an important regulator of both valve clusters with the highest expression of *Neo1* (coding Neogenin1) in cluster 4. Netrin signaling influences various signaling outputs such as cell adhesion, cell migration, and guidance as well as calcium influx via Piezo1.^34^^,^^35^^,^^36^ A better understanding of valve endothelial cell signaling and ion channel properties may provide new relevant targets for approaches in the treatment of valve insufficiency. In addition, we confirmed expression of genes involved in innate immune signaling and the negative regulation of coagulation in collecting lymphatic vessels, mentioned previously in single-cell analyses in ears and mesentery.^12^^,^^37^ Recent works have highlighted the crucial role of collecting lymphatic vessels in response to inflammation with the regulation of VCAM-1 and ACKR4 for efficient migration of immune cells to lymph nodes.^4^^,^^37^^,^^38^ Unlike their difference in term of structure and identity, capillaries and collecting lymphatic vessels have the capacity to sustain immune responses by the secretion of chemokines and upregulated adhesion molecules allowing selective migration of antigen-presenting immune cells.^4^^,^^12^^,^^13^^,^^37^^,^^38^ While we noticed a defined hierarchy of lymphatic vessel subtypes in the skin microenvironment, the comparison of the transcriptome of LECs from mesentery and dorsal skin shows similar molecular profiles of LECs despite their distinct organization and microenvironments.^39^ This suggests that at the transcriptional level, the microenvironment has little impact on the identity of LEC subpopulations, although their density may be more critical regarding the architecture and function of the tissues in which they reside. In depth analysis of LEC molecular mechanisms involved in translational control or post-translational protein modification may provide a better appreciation of LEC function and specialization. In summary, we found that 3D imaging of the entire lymphatic network will be valuable to address qualitatively and quantitatively lymphatic remodeling in skin diseases. Our work suggests that lymphatic valves may be more critical in forelimb and dorsal skin than in mesentery and ear skin. Future studies specifically designed to determine the role of lymphatic valve subpopulations might be beneficial to address how they contribute to lymphatic valve regulation and lymph drainage.

### Limitations of the study

3D imaging with reporter mouse models enables visualization of the lymphatic network and permits detailed quantification and analysis of lymphatic valve position. However, segmentation model is based solely on lymphatic architecture taking into account the density of initial capillaries but not the entire LYVE1-positive lymphatic capillary network. Moreover, precollecting vessels around hair follicles seem to have button junctions as capillaries. A quantification based on the presence of buttons junctions versus zipper junctions may give a better representation of the function of dermal lymphatic vasculature. Our single-cell analysis reveals an enrichment of genes involved in signaling pathways in LEC clusters, but we did not experimentally validate and functionally characterized these potential markers. While the localization of specific markers by immunostaining could help us to distinguish the two different valve clusters, these experiments are problematic due to a lack of antibody penetration and specificity in skin layers as well as difficulties associated with high resolution imaging of the two luminal leaflets in adult skin. Future studies are needed to validate the significance of the cellular mechanisms and signaling pathways revealed by our analysis.

## Resource availability

### Lead contact

Further information and reasonable request are available from the lead contact Florence Tatin: florence.tatin@inserm.fr.

### Materials availability

This study did not generate new unique materials.

### Data and code availability

• Raw data images generated in this study will be shared by the lead contact upon reasonable request.
• Single-cell dataset of LEC isolated from dorsal skin have been deposited at NCBI’s Gene expression Omnibus (GEO) under the accession number GEO: GSE295984 and are publicly available on the date of publication. The datasets analyzed in this study are available upon reasonable request from the corresponding author.
• The published single-cell dataset from *González-Loyola* et al.*, 2021* is publicly available from GEO with the accession number GSE156320.
• Any additional information required to reanalyze the data reported in this paper is available from the lead contact upon request.

## Acknowledgments

Our thanks go to the CREFRE US006 platform ANEXPLO Genotoul (Toulouse, France) and the Genomics and Transcriptomics platform of CRCT (Toulouse, France). We are grateful to the Genotoul bioinformatics platform Toulouse Occitanie (Bioinfo Genotoul) for providing help and/or computing resources. We acknowledge the imaging facility TRI, member of the national infrastructure France-Bioimaging, supported by the 10.13039/501100001665French National Research Agency, for helpful assistance on their field of expertise. This study was supported by funds from the 10.13039/501100001677National Institute for Health and Medical Research (10.13039/501100001677Inserm), France; University of Toulouse, France; European Regional Development Fund from Région Occitanie Pyrénées, France and 10.13039/501100004431Fondation de France, France.

## Author contributions

Conceptualization, E.M., F.T., and J.-M.L.; light-sheet acquisition, A.G., M.N., and P.B.-L.; image visualization and analysis, E.M., R.M., and F.T.; bioinformatic analysis, T.D.-N. and J.S.I.; writing, E.M., J.S.I., A. Briot, and F.T.; funding, F.T., B.G.-S., and A. Bouloumie. All authors contributed to the discussion of the manuscript.

## Declaration of interests

The authors declare no competing interests.

## STAR★Methods

### Key resources table

**Table:** 

REAGENT or RESOURCE	SOURCE	IDENTIFIER
**Antibodies**		

GFP Polyclonal Antibody [Alexa Fluor™ 647]	Invitrogen	Cat# A-31852; RRID: AB_162553
Perilipin Antibody [Alexa Fluor® 647]	Novus Biologicals	Cat# NB110-40760AF647; RRID:AB_3176698
DPPIV/CD26 Antibody	R&D System	Cat# AF954; RRID:AB_355739
αSMA (clone 1A4)	DAKO	Cat# M0851; RRID:AB_2223500
LYVE1 Antibody	R&D System	Cat# AF2125; RRID:AB_2297188
Alexa Fluor 546 anti-mouse	Thermo Fisher Scientific	Cat# A10036; RRID:AB_11180613
Alexa Fluor 594 anti-goat	Jackson ImmunoResearch	Cat#705-585-147; RRID:AB_2340433
CD45 PE-violet770	Miltenyi	Cat#130-110-661; RRID:AB_2658229
CD31-APC	Miltenyi	Cat#130-111-355; RRID:AB_2657299

**Biological samples**		

Mouse skin (forelimb and dorsal)	This paper	N/A
Isolated cells from Prox1-CreER^T2^;mTmG mouse	This paper	N/A

**Chemicals, peptides, and recombinant proteins**		

Liberase TM	Sigma	LIBTM-RO
Dispase II	Sigma	D4693-1G
RBC lysis buffer	Miltenyi	130-094-183
Donkey serum	Sigma	D9663
FcR blocking reagent CD16/CD32	Miltenyi	130-092-575

**Deposited data**		

Single-cell dataset	González-Loyola et al.^12^	GSE156320
Raw and analyzed data	This paper	GSE295984

**Experimental models: Organisms/strains**		

Mouse: *Prox1-CreER*^*T2*^	Taija Makinen; Bazigou et al.^40^	B6.Cg-Tg(Prox1-cre/ERT2)1Tmak; MGI:5617984
Mouse: *Vegfr3CreER*^*T2*^	Sagrario Ortega, CNIO; Martinez-Corral et al.^41^	Flt4tm2.1(cre/ERT2)Sgo; MGI: 5750213
Mouse: *R26-mTmG*	Beatrice Cousin, Restore UNIT; Muzumdar et al.^42^	Gt(ROSA)26Sortm4(ACTB-tdTomato,-EGFP)Luo; MGI: 3716464

**Software and algorithms**		

ImageJ	Schneider et al.^43^	https://imagej.nih.gov/ij/
ZEISS ZEN 3.3 (blue edition)	ZEISS	https://www.zeiss.com/microscopy/en/products/software/zeiss-zen.html#
IMARIS software	Oxford Instruments	https://imaris.oxinst.com/
GraphPad PRISM 9	GraphPad Software Inc.	https://www.graphpad.com/updates/prism-900-release-notes
Matlab	MathWorks	https://www.mathworks.com/products/matlab.html
Denoising algorithm	Buades et al.^44^	N/A
Thinning algorithm	Kollmannsberger et al.^45^	N/A
Adaptive Thresholding method	Bradley et al.^46^	N/A
3D Multipoint Annotation script	Remy Flores Flores	https://github.com/remyff/IJ_multi-point_length
R (version 4.4.3)		https://www.R-project.org
Seurat package (version 5.1.0)	Hao et al.^47^	N/A
Enrichr	Kuleshov et al.^48^	N/A
STRING functional protein association	Szklarczyk et al.^49^	N/A

**Other**		

FUnGI clearing protocol	Rios et al.^50^	N/A
BABB clearing protocol	Dierkes, Scherzinger, and Kiefer.^51^	N/A

### Experimental model and study participant details

#### Ethics statement

Animal experiments were conducted in accordance with recommendations of the European Convention for the Protection of Vertebrate Animals used for experimentation. All animal experiments followed procedures approved by the institutional local branch of the Midi-Pyrénées ethics committee and were performed according to the relevant guidelines for laboratory animal husbandry.

#### Mice

We used the *Prox1-CreER*^*T2*^ (kindly provided by Taija Makinen),^40^ *Vegfr3CreER*^*T2*^ (kindly provided by Sagrario Ortega, CNIO, Madrid)^41^ and *R26-mTmG*^42^ reporter mouse lines (kindly provided by Beatrice Cousin, Restore Unit, Toulouse). All strains were maintained on C57BL/6J genetic background in SOPF in Anexplo platform of Toulouse. Animals were group-housed in individually ventilated cages, with environmental enrichment, and had free access to food and water. Studies have been performed on female and male at 8 weeks old and 20 weeks old. For single-cells experiment, 3 males and 7 females from Prox1creERT2; mTmG mouse (*mTmg*^*lox/+*^*; Prox1creER*^*T2*^) littermates at 20 weeks aged were used (mean male weight: 32,7g; female weight: 24,3g). Animals were drug-naïve and had not undergone any prior procedures. For induction of Cre mediated recombination, mice were treated by daily administration of tamoxifen solution by oral gavage for 5 days (100μl at 10mg/mL in peanut oil). The light/dark cycle was 12 h, where the temperature and the humidity were stable at 19–23°C and 45–65%, respectively. The mice were anesthetized under isoflurane or xylasine/zoletil and euthanized by cervical dislocation.

### Method details

#### LEC isolation and cell sorting

Dorsal skin from 3 males and 7 females from *Prox1creER*^*T2*^*; mTmG* mouse at 20 weeks aged were collected 1 month after tamoxifen gavage (1mg/day during 5 days). Depilatory cream was used to remove hair from dorsal skin the day before under mouse anesthesia (xylasine/zolatil). Dorsal skin (around 2g) was placed in HBSS in 10cm dishes and transferred in digestion medium (1 U/mL Dispase II, 0,8 U/ml Liberase TM, 10U/mL DNase in Hanks’ Balanced Salt Solution solution) during 15 min at 37C. The dorsal skin was scraped and cell solution was incubated under agitation for 15 min at 37C. This two-step was repeated once and total cell solution was inhibited with DMEM with 10% FBS. Cell suspension was successively filtered through 100μm and 40um cell strainer, and incubated with RBC lysis buffer (eBioscience). Cells were then washed with FACS buffer and then incubated with conjugated antibodies CD45 PE-violet770 (130-110-661, Miltenyi) and CD31-APC (130-111-355, Miltenyi). FACS purified GFP-positive cells (CD31-APC positive and CD45-PE-violet770 negative) were isolated and processed through single cell Platform.

#### Immunostaining

Forelimb skin or dorsal skin were dissected, laid flat on a polymer gel and fixed overnight with 4% paraformaldehyde. Tissues were either used for paraffin sections or whole-mount immunostaining.

Mouse skin were embedded in paraffin. Tissue sections (6μm) were deparaffinized, antigen retrieval was achieved with a citrate-based buffer (10mM Sodium citrate in water, pH 6) at 95°C for 20 minutes. To block any residual active aldehyde groups, sections were incubated with 0.3M glycine solution, for 10 minutes, followed by blocking in PBS with 5% BSA for 2 hours. Primary antibodies diluted in PBS with 2% BSA were applied overnight at 4°C in a humidified chamber. Excess primary antibodies were washed 3 times in PBS, and secondary antibodies and DAPI diluted in PBS with 2% BSA were applied 1 hour at room temperature in humidified chamber. After 3 PBS washes, sections were mounted with Mowiol 4-88 (Sigma, 81381).

For wholemount staining, samples have been processed as described in Dierkes C. et al, 2018.^51^ Briefly, samples were fixed in PFA 4% at 4°C overnight. Then, samples were washed 3 times in PBS+NaN3 0.5%, permeabilized in Triton 0,5% at 4°C during 1 days. After 3 times of washing solution (PBS with 0.1% Tween), samples were incubated with Permblock solution (1% BSA, 0,5% Tween, 5% donkey serum, in PBS+NaN3) at 4°C during 1 days. Then, samples were in solution with primary antibody diluted in Permblock buffer during 3 days in a 96 or 24 well-plate. After a washing step during 1 day, changed every hour, secondary antibodies diluted in Permblock were added during 3 days. The following primary antibodies were used: anti-GFP polyclonal antibody conjugated with Alexa Fluor 647 (A-31852, Invitrogen), rabbit anti-Perilipin polyclonal antibody conjugated with Alexa Fluor 647 (NB110-40760AF647, Novus Biologicals), goat anti-DPP4 (AF954, R&D system), mouse anti-human αSMA (clone 1A4, M0851, Dako), goat anti-LYVE1 (AF2125, R&D system). Secondary antibodies Alexa Fluor 546 anti-mouse or Alexa Fluor 594 anti-goat were used (Invitrogen and Jackson Immunoresearch respectively).

#### Tissue clearing

Tissue clearing for the preservation of GFP expression followed by Two-photon microscopy (Figure S2D) has been performed as described.^50^ Samples were immersed in FUnGI Solution (50% glycerol (vol/vol), 2.5M fructose, 2.5M urea, 10mM Tris Base, 1.0mMEDTA) for at least 3h. BABB clearing was used for all samples with light-sheet microscopy. After staining, samples were dehydrated in methanol (50%, 70%, 95% and 2 times 100%, each step 30 min). Then, samples were optically cleared in solution with 50% BABB/ 50% methanol for 5h (Benzyl alcohol: benzyl benzoate with a ratio of 1:2) and transferred in 100% BABB solution.

### Quantification and statistical analysis

#### Light-sheet and confocal microscopy

Image acquisitions were done using a Zeiss Light-sheet Z.1 microscope (Imactiv-3D) at different magnifications with objective 2,5X: Figure 1C (volume image is 3,51mm x 3,51mm x 495μm with a step of 5 μm), Figure S2A (volume image is 8,61mm x 15,98mm x 3,35μm with a z-step of 10μm), Figure S2B (volume image is 2,7mm x 3,1mm x 1mm with a z-step of 10μm) and Figure S2C (3,51mm x 3,51mm x 2.07mm with a step of 6.52μm). With objective 5X: Figure 1D (volume image is 1,76mm x 1,76mm x 315μm with a z-step of 5μm), Figure S3A (volume image of 1,692mm x 1,692mm x 298μm with a z-step of 10μm. With objective 20X: Figure 2A (magnification from initial image of 421,18μm x 421,18μm x 628 μm with a z-step of 1μm).

Paraffin sections were acquired using Confocal microscope LSM900. Representative 3D-images of the z-stacks were done using Zen software (Zen 3.3), Image J software^43^ or Imaris software.

#### Image processing and quantification

Image analysis for the characterization of the murine lymphatic network was performed using an image processing pipeline developed in Matlab which consists of the following stages (Figure S3).

A 3D region of interest (ROI) was first manually extracted from each image acquired according to various features, e.g., image quality and content relevance. The ROIs were designed according to an individual approximate volume of 1 mm^3^ and were resampled in order to produce upscaled isotropic images (spatial resolution = 1.8 μm). Detection of the entire lymphatic network was then obtained using a preconditioning 3D image denoising algorithm derived from non-local means denoising^44^ followed by a 3D hysteresis segmentation step including an underlying dual thresholding operation. Any residual connected component whose volume was inferior to 10^6^ voxels (5.8 x 10^6^ μm^3^) was considered as an artefact and discarded. From the 3D segmentation image, a 3D skeleton was computed using a parallel medial axis thinning algorithm^45^ based on an optimized homotopic thinning implementation. The skeleton was in turn converted into a network graph described by its nodes, edges and adjacency matrix. The graph conversion operation was repeated until convergence of the number of nodes and edges to ensure accurate connectivity. The threshold for the minimum length of edges was set to 10 μm to improve computational load. Once the network graph was generated, the network was split into two components, namely branches and links depending on whether they were exiting edges or not. They correspond to capillaries and pre-collectors, respectively. All the network’s elements connected to the ROI border were either considered as cropped capillaries (length inferior to 25 μm) and discarded from further analysis or as cropped precollector (length superior to 300 μm).

Based on the 3D segmentation of the entire network, valves were detected inside the regions defined by the network’s mask by a further segmentation scheme. In the first place, residual background signals are attenuated using morphological opening and a spherical structural element of 18 μm in radius. An adaptive binarization method^46^ based on local first-order image statistics around each pixel was then implemented in order to cope with the strong intensity heterogeneity of the valves. Sensitivity was set to 0.5. In order to reduce false positive detection rate, a Gaussian blurring filtering was performed beforehand (sigma = 2) and only connected components whose volume was between 1 x 10^3^ μm^3^ and 1 x 10^4^ μm^3^ were classified as acceptable valves. Various parameters were computed to characterize the lymphatic network: network density in terms of volume (volume of network / volume of sample) and length (length of network / volume of sample), minimum and maximum z-position of the capillaries, length of all the components (capillaries and pre-collectors) of the network, as well as 3D orientation of the capillaries (computed as the orientation of the ellipsoid that has the same normalized second central moments as each capillary, and interpreting the angles by looking at the origin along the x-, y-, and z-axis representing roll, pitch, and yaw respectively).

For the analysis of the distance between lymphatic valves using 3D Multipoint Annotation: To quantify the geometry of biological structures in three-dimensional images, a custom script (https://github.com/remyff/IJ_multi-point_length) was developed in Fiji (ImageJ). This script computes two morphometric parameters from manually placed multipoint annotations: cumulative length and range. Points are placed using ImageJ “Multi-point” tool to follow the shape of a structure in 2D or 3D. Upon execution, the script adds the current selection to the ROI Manager (for record keeping), computes the distances, logs the measurements in the Results Table, and clears the points from the active image. The length is calculated as the sum of the Euclidean distances between each consecutive pair of points, accounting for voxel dimensions in both XY and Z. The range is defined as the Euclidean distance between the first and last points of the annotation.

#### Single-cell analysis

Single-cells were captured using the Chromium Controller and the Chromium Next GEM single cell 3' v3.1 kit (10x Genomics, Inc.). Dual-indexed single-cell RNAseq libraries were prepared according to the manufacturer’s instructions. The library was profiled with the HS NGS kit for the Fragment Analyzer (Agilent Technologies) and quantified using the KAPA library quantification kit (Roche Diagnostics). The library was sequenced on the Illumina NextSeq 500 instrument using the High Output 150 cycles kit (Illumina). The sequencing parameters were 28, 10, 10, 90 cycles (read 1, index 1, index 2, read 2). A minimum of 125,406 mean reads/cell were obtained. STAR (version 2.7.11a^52^) was used to align fastq to the mouse genome (Gencode M32/GRCm39^53^), with the following parameters: --limitOutSJcollapsed 200000000, --soloType CB_UMI_Simple, --soloUMIlen 12, --soloCBwhitelist 3M-february-2018.txt, --soloFeatures “Gene GeneFull SJ Velocyto”, --soloCellFilter EmptyDrops_CR. Gene/filtered outputs were read into R (version 4.4.3; https://www.R-project.org) with the Seurat package’s (version 5.1.0^47^) ReadSTARsolo function. Cells were select with percent mitochondrial reads between 0.5% and 5.5% and number of features between 250 and 2500. After normalization, the mean.var.plot selection method was used with FindVariableFeatures to obtain 2000 genes for scaling, PCA and ICA with 30 dimensions. FindMultiModalNeighbors was used with the PCA and ICA reductions to obtain a weighted.nn for RunUMAP and wsnn for FindClusters (algorithm = 1, resolution = 0.25).

Prox1 expression was used as a marker for lymphatic cells and 6 distinct clusters were identified with a resolution of 0.45 during clustering. FindAllMarkers was used to find cluster specific marker genes. The top 10 genes per cluster based on the difference between pct.1 and pct.2 were presented as a heatmap with DoHeatmap and selected marker genes were presented in violin plots with VlnPlot, all from the Seurat package. All UMAP plots were generated with Seurat’s DimPlot or FeaturePlot functions.

Enrichr^48^ was used with the Gene Ontology database (http://geneontology.org) to attribute functional pathways to cluster specific marker genes. Select genes from select pathways were presented as spot plots with the Seurat DotPlot function.

ggplot2 and ggrepel (https://doi.org/10.32614/CRAN.package.ggrepel) were used for the x/y plots. All colors were selected from RColorBrewer palettes (RColorBrewer ColorBrewer Palettes. R Package Version 1.1-3). STRING functional protein association was used to visualize distinct signaling pathway.^49^

#### Analysis of publicly available scRNA-Seq data

Single-cell dataset from *González-Loyola et al, 2021* available in NCBI’s Gene expression Omnibus (GEO) accession number GSE156320 was used. Only raw fastq data from WT mouse mesenteries were analysed.

Fastq files from GSE156320, GSM4716880 and GSM4716881 respectively, were obtained from the Gene Expression Omnibus and aligned with STAR as described above. Subsetting was done to retain 1250 to 3500 for number of features and 0.25 to 5.0 for mitochondrial percentage. The pipeline above was used for initial exploration of each GSM and then Seurat was used to perform integration as follows. We combined the three dataset into layers, found variable features for each dataset with the mean.var.plot method, obtained a PCA with 30 dimensions and ran IntegrateLayers with CCA, RPCA and Harmony integration methods based on the PCA. These three integrations were used with FindMultiModalNeighbors to obtain a weighted.nn for RunUMAP and wsnn for FindClusters (algorithm = 1, resolution = 0.25). Seurat’s FindAllMarkers was used on the integrated clusters to find cluster markers in each dataset separately and geom_alluvium from the ggalluvial package was used to make the Alluvial plot depicting the overlap of cluster markers between the skin cells from this study and the mesentery cells from GSE156320 (R package version 0.12.5 ; http://corybrunson.github.io/ggalluvial).

#### Statistical analysis

Statistical analyses were performed using GraphPad prism 9 software. Significance was analysed using ANOVA. Sample size and p values are indicated in each figure legend by asterisks∗.
