# Supplementary material for: 3D imaging and single-cell analysis reveal cellular heterogeneity of lymphatic valve endothelial cell types
Source: iScience. 2025 Oct 24;28(11):113841. doi: 10.1016/j.isci.2025.113841 (PMC12648505; doi:10.1016/j.isci.2025.113841)
Supplement: Document S1. Figures S1–S5 [file mmc1.pdf]

iScience, Volume *28*

Supplemental information

3D imaging and single-cell analysis reveal cellular heterogeneity of lymphatic valve endothelial cell types

Emmanuelle Marchaud, Renaud Morin, Jason S. Iacovoni, Tangra Draia-Nicolau, Aurélie Gomes, Marine Norlund, Pascale Bernes-Lasserre, Jean-Michel Lagarde, Anne-Catherine Prats, Barbara Garmy-Susini, Anne Bouloumie, Anaïs Briot, and Florence Tatin

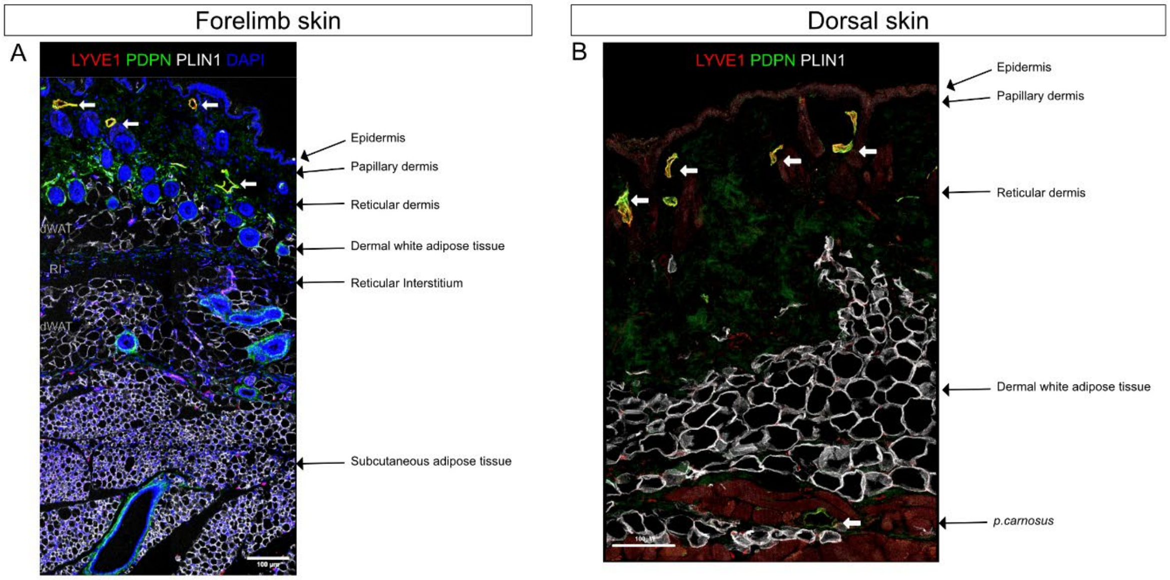

Figure S1: Conventional 2D-imaging does not allow visualization of lymphatic vessel architecture in skin. Related to Figure 1.

(A) Confocal image of immunostained paraffin section (5 µm) from forelimb skin of 8 week-old mouse showing a lymphatic capillary (PDPN+ and high LYVE1+, green and red, resp.) and a lymphatic pre-collector (PDPN+ and low LYVE1). (B) Confocal image of immunostained paraffin section (5 µm) from dorsal skin of 20 week-old mice showing a lymphatic capillary (PDPN+ and high LYVE1+, green and red, resp.). Note the presence of a lymphatic vessel in proximity to dermal adipose and muscle layer (white arrow). Scale bar: 100 µm.

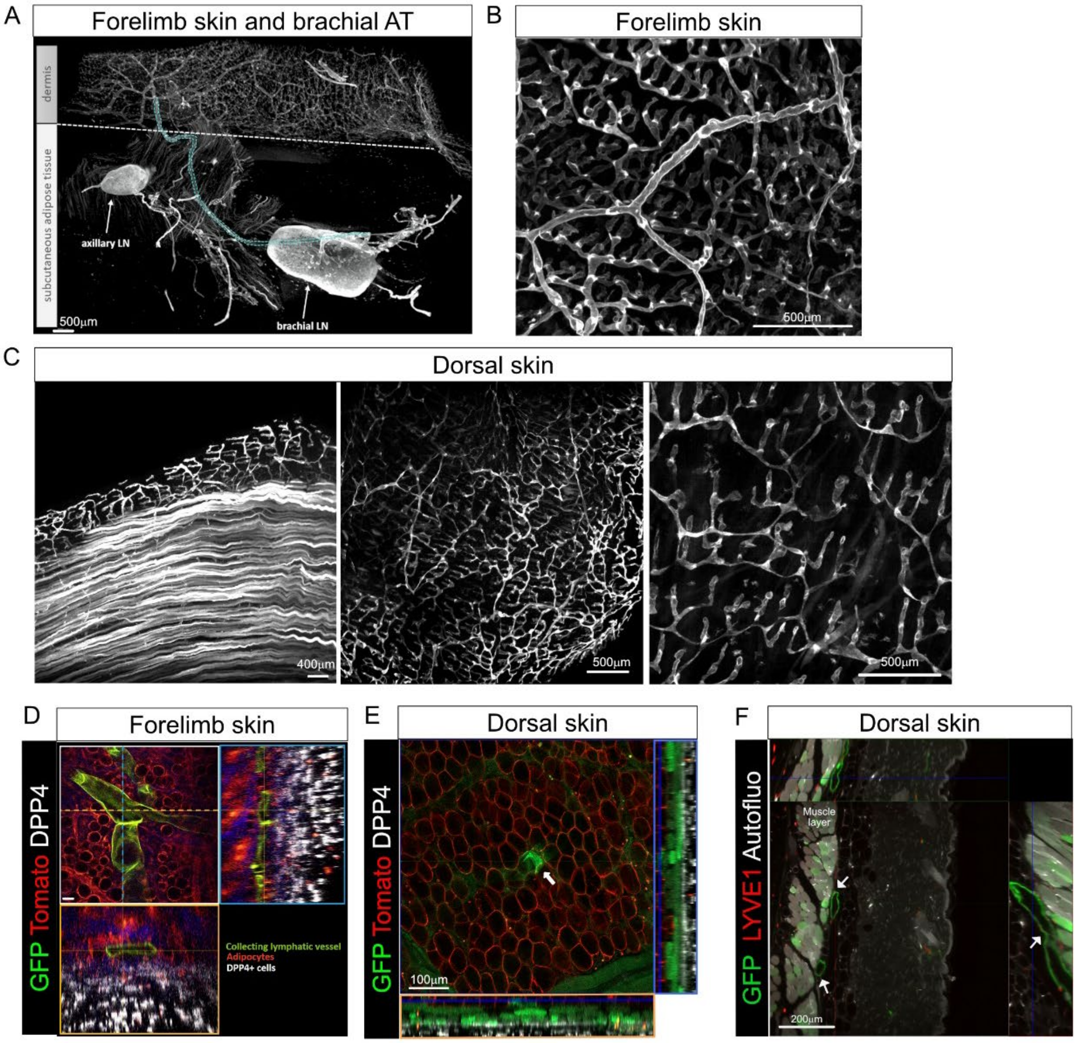

Figure S2: Architecture of lymphatic vasculature in forelimb and dorsal skin. Related to Figure 1.

(A) Three-dimensional image reconstruction of the lymphatic vessels in forelimb skin of *Vegfr3-creER^{T2}; R26-mTmG* and its subcutaneous adipose tissue. The lymphatic network is mainly located in the dermis, and a collecting vessel (framed by cyan dotted lines) is connecting this dermal zone to the brachial lymph node in subcutaneous adipose tissue. Note the density of lymphatic network in dermis compared to subcutaneous adipose tissue. Scale bar: 500 µm. (B) Magnified view of the lymphatic network in dermis. Note the well-established organization of this lymphatic network and numerous lymphatic valves. Scale bar: 500 µm. (C) Three-dimensional image of dorsal skin of *Prox1-creER^{T2}; R26-mTmG* mice with and without the muscle layer to better visualize the lymphatic network. Magnified view of the lymphatic network is shown on the right panel. Scale

bar: 400 µm. D) Orthogonal projection of collecting vessels within the adipocytes (Tomato expression in red) and close to a layer of DPP4+ progenitor cells delimiting the reticular interstitium in arm skin. Scale bar: 30 µm. E) Orthogonal view of lymphatic vessels (GFP+, white arrows) in dermal adipose layer above the muscle layer visible here in GFP channel Note that the DPP4+ reticulum interstitium is located under the muscle layer in dorsal skin. Scale bar: 100 µm. F) orthogonal view of collecting lymphatic vessels in the dermal adipose layer in proximity to muscle. Scale bar: 200 µm.

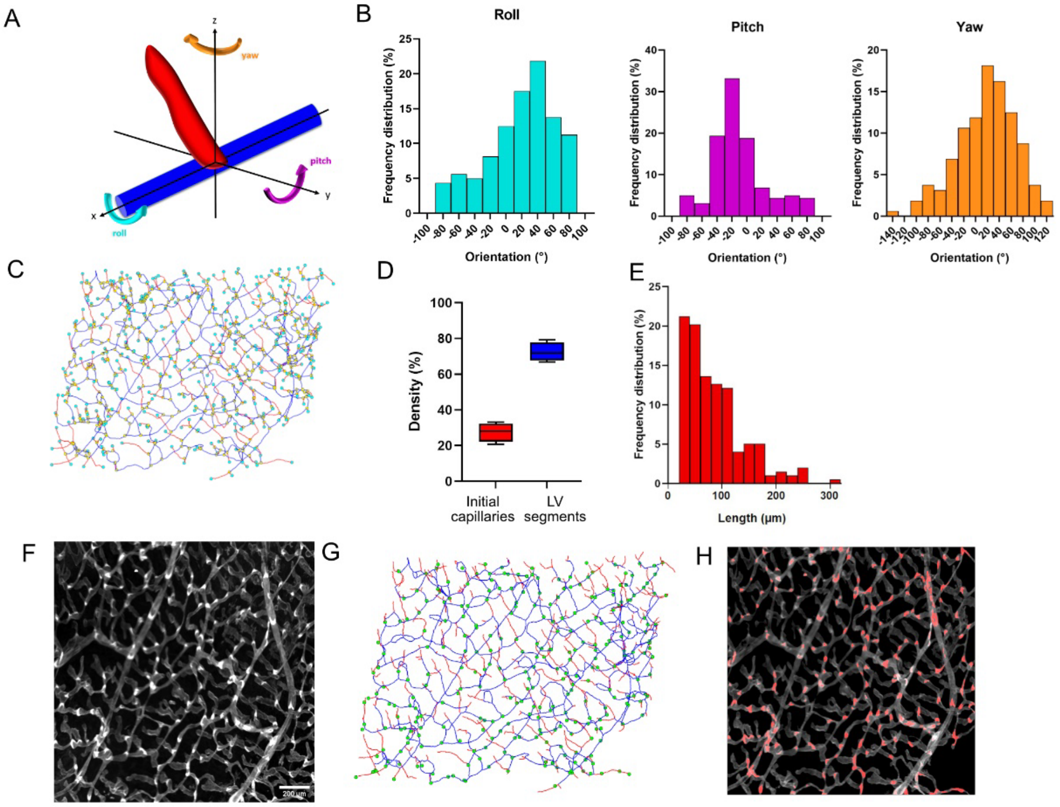

I

Tissues	Sample volume [mm³]	Network density [mm/mm³]	Initial capillary number	Initial capillary length [mm]	Initial capillary density [mm/mm³]	LV segment number	Total LV length [mm]	LV density [mm/mm³]
**1**	1,1	54,0	198	17,1	16,1	497	40,3	37,9
**2**	1,0	68,7	235	13,7	14,3	874	52,0	54,4
**3**	1,5	56,9	432	27,3	18,8	1031	55,2	38,1
**4**	1,3	62,7	372	22,2	16,5	1265	61,8	46,1

Figure S3: Quantitative analysis of the lymphatic network using 3D images. Related to Figure 1.

(A) Schematic representation for the characterization of the 3D-orientation of capillaries in the skin. The plane formed by the precollectors, which are parallel to the epidermis, forms the x,y plane. The orientation of a capillary in 3D is composed of 3 angles: roll, pitch and yaw along the x-, y- and z-axis respectively. (B) The frequency distribution for each orientation is reaching a pic, suggesting that capillaries follow an orientation through all the sample. For example, 33% of the capillaries follow an orientation between -30° and -10° along the y-axis. (C) 3D-segmentation image allows to visualize a network graph described by nodes (yellow) and edges (cyan). (D) Histogram showing the percentage of the density of initial lymphatic capillaries compared to others lymphatic segments (n=4). (E) Histograms showing the length of initial capillaries within the skin.

(F) The region of interest is defined as a crop of 1.1mm3 extracted from the total acquisition to characterize the lymphatic network in the skin of *Prox1-creER^{T2}; R26-mTmG mice*. Scale bar: 200 µm. (G) Segmented network graph showing the position of lymphatic valves as green nodes. (H) Detection of valves (shown in red) reported on the lymphatic network. Scale bar: 200 µm. (I) Recapitulative table of the quantifications processed from light-sheet microscopy acquisitions from 4 different tissues.

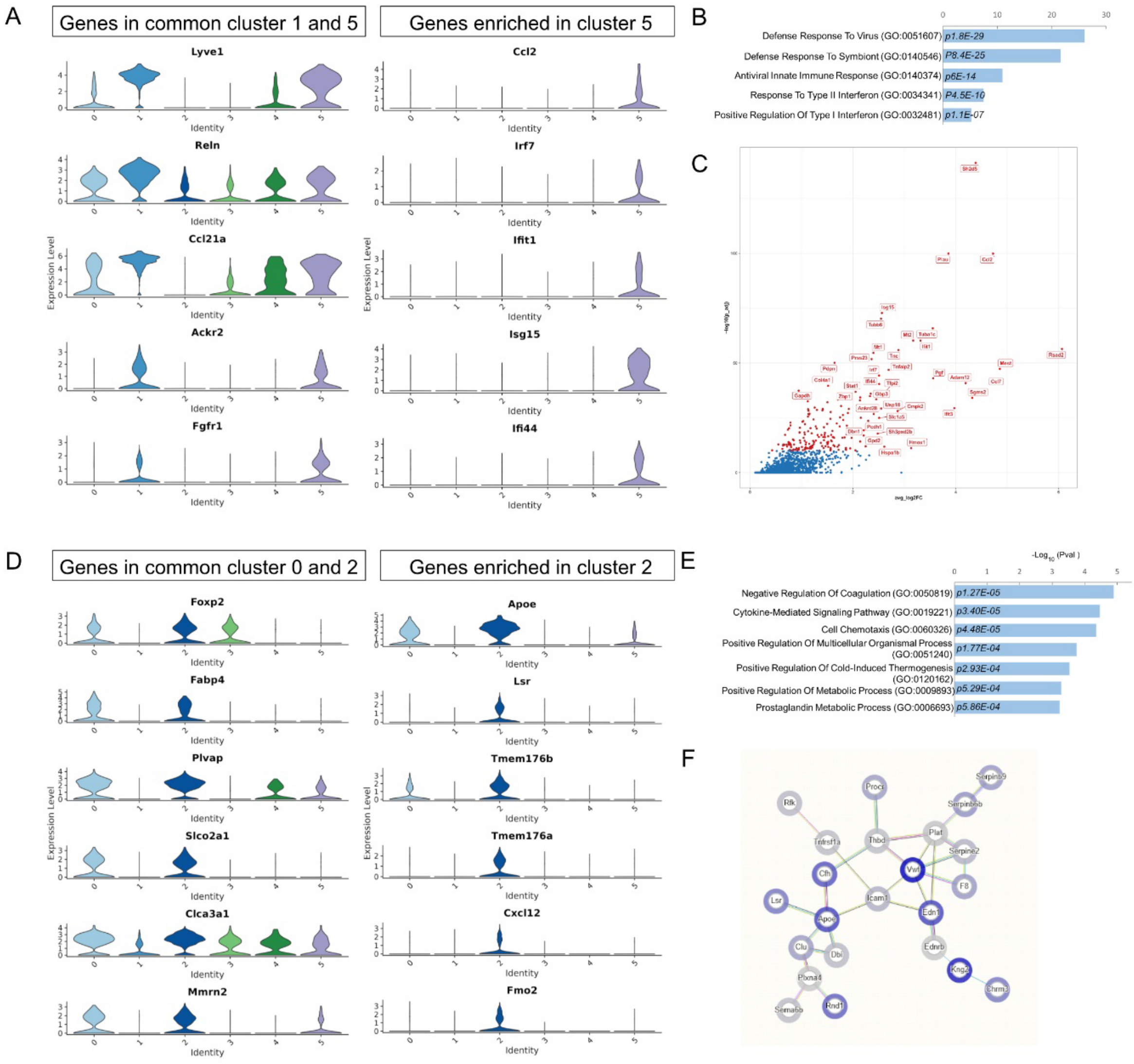

Figure S4: Characterization of LEC diversity. Related to Figure 3.

A) Violin plot of genes shared in common by capillaries and unique to cluster 5. B) Bar plots representing the 5 most enriched GO signaling among genes up-regulated in cluster 5. C) Volcano plot showing gene expression (logFC) versus statistical significance. D) Violin plot of genes shared in common by cluster 0 (precollecting vessels) and cluster 2 (collecting vessels) and enriched in cluster 2. Note specific genes involved in immune signaling are enriched in collecting lymphatic vessels. (E) Bar plots representing the 5 most enriched GO signaling among genes up-regulated in cluster 2 (collecting vessels). F) Predictive protein associations network among genes up-regulated in cluster 2 (Selected genes with logFC>0.5, dpct>10%) (STRING database, setting with high confidence>0.7).

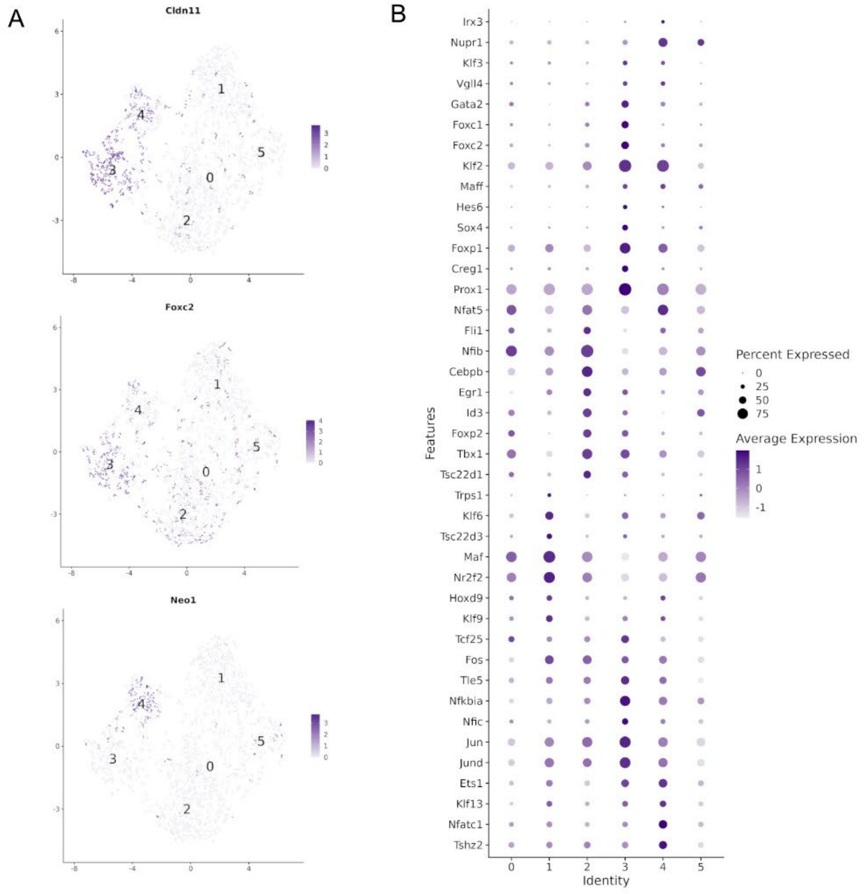

Figure S5: Specific signature of lymphatic valve subpopulations. Related to Figure 3.

A) UMAP of known markers to classify lymphatic capillaries, precollecting and collecting vessels and lymphatic valves clusters. B) Heatmap of main transcription factors and co-regulators in different LEC subpopulations. Note the enrichment in *Irx3*, *Nupr1*, *Nfat5*, *Ets1*, *Klf3*, and *Nfatc1* in cluster 4 compared to other clusters.
